# Identification of the Avian *Pasteurella multocida phoP* Gene and Evaluation of the Effects of *phoP* Deletion on Virulence and Immunogenicity

**DOI:** 10.3390/ijms17010012

**Published:** 2015-12-23

**Authors:** Kangpeng Xiao, Qing Liu, Xueyan Liu, Yunlong Hu, Xinxin Zhao, Qingke Kong

**Affiliations:** 1 Institute of Preventive Veterinary Medicine, College of Veterinary Medicine, Sichuan Agricultural University, Chengdu 611130, China; s20123512@stu.sicau.edu.cn (K.X.); s20123525@stu.sicau.edu.cn (X.L.); huyunlong@sicau.edu.cn (Y.H.); 2 Department of Bioengineering, College of Veterinary Medicine, Sichuan Agricultural University, No. 211 Huimin Road, Wenjiang District, Chengdu 611130, China; qing.liu.2@sicau.edu.cn; 3 Avian Disease Research Center, College of Veterinary Medicine, Sichuan Agricultural University, Wenjiang, Chengdu 611130, China; 4 Key Laboratory of Animal Disease and Human Health of Sichuan Province, Sichuan Agricultural University, Wenjiang, Chengdu 611130, China

**Keywords:** *Pasteurella multocida*, phoP, virulence, regulated genes, vaccine

## Abstract

*Pasteurella multocida* (*P. multocida*) is an animal pathogen of worldwide economic significance that causes fowl cholera in poultry and wild birds. Global gene regulators, including PhoP are important in regulating bacterial virulence and are good targets for developing attenuated vaccines against many pathogenic bacteria. However, the biological significance of *phoP* gene has not been identified in *P. multocida*. Here, we identified the *phoP* gene in *P. multocida*, and we evaluated the roles of *phoP* in *P. multocida* by deleting the *phoP* gene. The *P. multocida phoP* mutant exhibited similar growth curves and lipopolysaccharide and outer membrane protein profiles but displayed defective polymyxin resistance *in vitro* compared with the parent strain. Additionally, the *phoP* deletion resulted in decreased virulence. The LD_50_ of the Δ*phoP* mutant was 32- and 154-fold higher than the parent strain via the oral and intranasal routes, respectively. Transcriptome sequencing analysis showed that 161 genes were up-regulated and 173 genes were down-regulated in the absence of the *phoP* gene. Finally, the immunogenicity and protective efficacy of the Δ*phoP* mutant were evaluated. Immunized ducks produced significantly higher levels of serum IgY and bile IgA compared to the control ducks, and immunization with the Δ*phoP* mutant conferred 54.5% protection efficiency against challenge with the virulent *P. multocida*. This work provides a platform to dissect the function of *phoP* and develop a new vaccine against *P. multocida*.

## 1. Introduction

*Pasteurella multocida* (*P. multocida*) is a Gram-negative encapsulated bacterium that is the causative agent of a range of animal pasteurellosis diseases, including fowl cholera in poultry and wild birds, hemorrhagic septicemia in cattle and buffalo, atrophic rhinitis in swine, and snuffles in rabbits [1]. *P. multocida* can also infect human via small-mammal bites. Fowl cholera is a severe systemic disease that occurs in domestic poultry and wild birds and results in significant economic losses to poultry industries worldwide [2]. The use of antibiotics is a successful method of controlling *P. multocida* infection, but the emergence of drug-resistant strains poses a serious challenge for antibiotic use [3]. Vaccination of fowl would provide protection against *P. multocida* infection [4]. Current vaccines against fowl cholera include bacterins and live attenuated vaccines: the former provides only limited protection against homologous serotypes, and the latter were developed empirically and were observed to revert to the virulent strain [4]. Therefore, there is a pressing need to develop new vaccines, especially well-defined live vaccines for fowl cholera control.

Live attenuated vaccines were successfully developed by the introduction of mutations into global regulators including two-component systems (TCS) in many pathogenic bacteria [5,6]. TCS are highly conserved prokaryotic signal transduction pathways that consist of a histidine kinase as the sensor and a response regulator as the effector. Intracellular pathogens usually use TCS to respond to host defenses and are often essential for virulence; the *Salmonella* *phoP* is the most widely studied example [6]. *phoP* was first identified and suggested to perform a regulatory function in *Salmonella* because a strain with a *phoP* mutation lost acid phosphatase activity [7]. *phoP* and *phoQ* constitute a vital TCS involved in bacterial invasion and survival in the host and can positively and negatively regulate a network of genes in many Gram-negative pathogens, including *Mycobacterium tuberculosis* [8], *Yersinia pestis* [9], *Escherichia coli* (*E. coli*) [10], and *Salmonella enterica* [11]. In response to a number of environmental signals, including low magnesium [12], low pH [13], and sub-lethal cationic antimicrobial peptides [14], PhoQ phosphorylates PhoP, which regulates the expression of genes containing an 18-bp PhoP box sequence within their promoters [15]. Activation of this operon leads to modifications of lipopolysaccharides (LPS) or outer membrane proteins (OMPs) to cope with environmental extremes and enhance bacterial survival [16]. Deletion of *phoP* significantly decreases the virulence of some bacteria [17], and increases the sensitivity to antimicrobial cationic peptides such as polymyxin B [18] and the *phoP* mutant strain also confers protective immunity against challenge with pathogenic bacteria [19,20,21], indicating that the *phoP* gene would be an ideal target for developing an attenuated live vaccine.

During infection, *P. multocida* encounters a wide-range of adverse environments including dramatic shifts in nutrient acquisition, pH, iron and innate immune molecules such as reactive oxygen species, antimicrobial peptides and those found in the immune cells [22]. Overcoming these adverse limitations are essential for establishing a *P. multocida* infection in the host. *P. multocida* may exploit a similar TCS mechanism as *Salmonella* to sense the exterior signals. The sequence and functions of the *phoP* gene in *P. multocida* are not known. In this study, the *phoP* gene was first cloned from the highly virulent *P. multocida* 0818 strain isolated from ducks in southwest China. A non-polar Δ*phoP* mutant of *P. multocida* 0818 was then constructed and was systemically investigated for several phenotypes, including bacterial growth, LPS and OMP profiles, resistance to polymyxin B, and virulence in ducks. The *phoP*-regulated genes were also identified by transcriptome sequencing. Moreover, the immunogenicity and protective efficacy of the Δ*phoP* mutant were determined.

## 2. Results and Discussion

### 2.1. Results

#### 2.1.1. Cloning and Characterization of the *phoP* Gene of *P. multocida*

**Figure 1:**
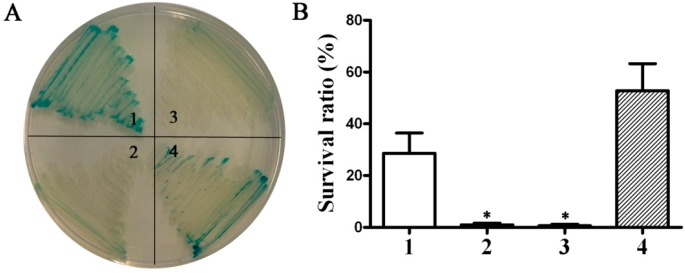
Identification of the *phoP* gene of *P. multocida*. (**A**) Detection of phosphatase activity. Four *S.* Typhimurium strains, S184 (Δ*asd*) harboring empty plasmid pQK664, S412 (Δ*asd* Δ*phoP*) harboring pQK664, S412 harboring pQK167 and S412 harboring pQK168, were cultured on X-P plates supplemented with 50 μg/mL diaminopimelic acid (DAP), and the colony colors were observed; (**B**) The resistance to polymyxin B. The four strains described in A were cultured in Luria-Bertani (LB) broth with or without 0.1 μg/mL polymyxin B for 1 h, and then the survival ratio of each strain was calculated. 1, 2, 3 and 4 refer to S184 (pQK664), S412 (pQK664), S412 (pQK167) and S412 (pQK168), respectively. The data in **B** are expressed as the mean value ± standard deviation (SD) and were analyzed with the significance level set at 0.05 (*) compared to the S184 strain.

Based on the bioinformatics analysis, two potential *phoP* genes, named *phoP1* (PM0432, NCBI Gene ID: 1243779) and *phoP2* (PM0214, NCBI Gene ID: 1243561), were cloned from *P. multocida*. The proteins encoded by the *phoP1* and *phoP2* gene shared 27.23% and 36.53% identity with the PhoP of *Salmonella enterica* serovar Typhimurium (*S.* Typhimurium), respectively*.* To study the cloned genes, the recombinant Asd^+^ (aspartate semialdehyde dehydrogenase) plasmids pQK167 (*phoP1*) and pQK168 (*phoP2*) were transformed into S412 (*S.* Typhimurium Δ*asd* Δ*phoP*). Phosphatase activity and the resistance to polymyxin B were determined in S184 (*S.* Typhimurium Δ*asd*) harboring the empty plasmid pQK664, S412 harboring pQK664, S412 harboring pQK167 and S412 harboring pQK168. Asd is required for the synthesis of diaminopimelic acid (DAP), an essential component for biosynthesis of cell walls in *Salmonella*. It has been demonstrated that *S.* Typhimurium is incapable of survival in the absence of DAP *ex vivo*. A balanced-lethal host-vector system based on the essential bacterial gene for Asd has been used to specify recombinant proteins from Asd^+^ plasmids that are retained in *Salmonella* strains with the *asd* gene deleted [23,24]. Here, the aim of selecting Δ*asd* strains for identifying the potential *phoP* gene was to stably maintain the exogenous Asd^+^ plasmids in the bacteria without antibiotic pressure. As shown in Figure 1A, the S412 strain displayed a white colony morphology and S184 showed blue colony morphology on X-P plates, indicating that *phoP* deletion leads to suppressed phosphatase activity [14]. Meanwhile, S412 harboring pQK168 (*phoP*2) rather than pQK167 (*phoP1*) displayed a similar phenotype (blue clones) as strain S184 (Figure 1A). The survival ratio of S184 after treatment with polymyxin B was significantly higher than that of S412, which lacked the *phoP* gene (Figure 1B). The plasmid pQK168 (*phoP2*), but not pQK167 (*phoP1*), had the ability to restore resistance of S412 to polymyxin B (Figure 1B), indicating that the *phoP2* gene of *P. multocida* could complement the *phoP* gene mutation in *S.* Typhimurium. Both the phosphatase assay and the polymyxin B resistance assay demonstrated that *phoP2* was the functional *phoP* gene in *P. multocida.*

**Figure 2:**
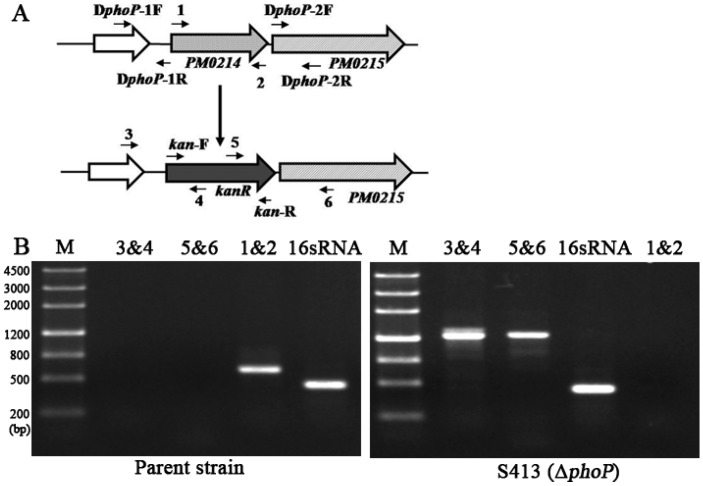
Construction of the Δ*phoP* mutant in *P. multocida* strain 0818. (**A**) Schematic strategy for deletion of the target *phoP* gene (PM0214) by single-crossover recombination. The targeted *phoP* gene of *P. multocida* 0818 was replaced with a *kanR* cassette through homologous recombination. The primers termed 1, 2, 3, 4, 5 and 6 were designed for selection and characterization of the mutant clones; (**B**) Characterization of the constructed Δ*phoP* mutant. The parent strain and the Δ*phoP* mutant were identified with three primer pairs: 1&2, 3&4 and 5&6. The theoretical sizes of the PCR products from primer pairs of 1&2, 3&4, 5&6 and 16sRNA-F/R were 672, 1168, 1282 and 501 bp, respectively. M refers to the DNA marker; 16S RNA denotes amplification of the positive-control gene in both strains.

#### 2.1.2. Construction of the Non-Polar Δ*phoP* Mutant in *P. multocida* 0818

To determine the roles of the *phoP* gene in *P. multocida* 0818, a *P. multocida* Δ*phoP* mutant named S413 was constructed through suicide plasmid-mediated homologous recombination. The target *phoP* (PM0214) gene was replaced with the *kanR* (kanamycin resistance) cassette by single-crossover recombination (Figure 2A). The mutant was selected and purified by kanamycin pressure and was confirmed by PCR amplification with three primer pairs (1&2, 3&4 and 5&6) (Figure 2A). As shown in Figure 2B, the fragment containing *phoP* upstream sequence and partial *kanR* cassette sequence, or *phoP* downstream sequence and partial *kanR* cassette sequence, was amplified from strain S413 but not the parent strain (*P. multocida* 0818), and the complete *phoP* gene was only amplified from the parent strain. The positive-control 16S rRNA gene was present in both strains. Thus, the *phoP* gene was successfully deleted in S413.

#### 2.1.3. Phenotype of the Δ*phoP* Mutant Strain

**Figure 3:**
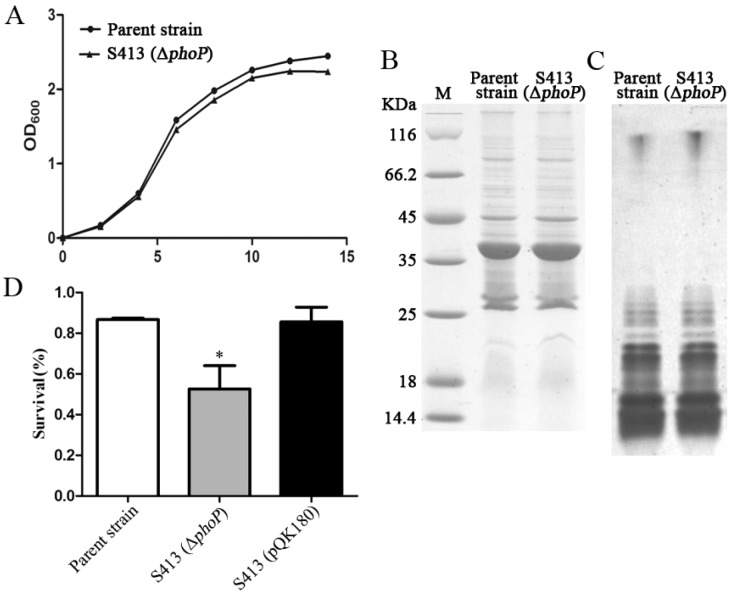
Phenotype characterization of the *P. multocida* Δ*phoP* mutant. (**A**) The growth curve of the parent strain and the Δ*phoP* mutant. *P. multocida* were cultured in brain heart infusion (BHI) broth supplemented with or without kanamycin, and then the OD_600_ value of each strain was measured every 2 h over a period of 14 h; (**B**,**C**) The OMP and LPS profiles of *P. multocida*. OMPs or LPS were extracted from the parent strain or the Δ*phoP* mutant and were then subjected to sodium dodecyl sulfate polyacrylamide gel electrophoresis (SDS-PAGE). Next, Coomassie blue staining and silver staining were applied to visualize the OMPs (**B**) and LPS (**C**), respectively. M refers to the protein Marker; (**D**) The resistance to polymyxin B. The parent strain, S413 and the complement stain S413 (pQK180) were cultured in BHI broth with or without 0.5 μg/mL polymyxin B for 1 h, and the survival ratio of each strain was calculated as the mean CFU of the polymyxin B-treated group divided by the mean CFU of the untreated group. The data in **D** are expressed as the mean ± SD and were analyzed at the significance level of 0.05 (*).

To evaluate effects of the *phoP* mutation on the *P. multocida* phenotypes, the growth curve, the OMPs and LPS profiles and the resistance to polymyxin B were measured. Strain S413 (*P. multocida* Δ*phoP*) and the parent strain showed similar growth curves with a short lag phase (0–2 h) and a log phase where major bacterial growth occurred (2–10 h) followed by a stationary phase (10–14 h) (Figure 3A). The OMP and LPS bands were similar in both strains and were mainly composed of proteins larger than 25 kDa and short-length LPS, respectively (Figure 3B,C); however, subtle differences in OMPs or LPS might be present that can not detected by the Coomassie blue or silver staining. In contrast, the polymyxin B resistance significantly decreased in the absence of *phoP*. The survival ratio of strain S413 after treatment with polymyxin B was significantly lower than that of the parent strain and the complement strain, S413 with pQK180 carrying the *phoP* gene of *P. multocida* (Figure 3D). The successful complementation by pQK180 also demonstrated the non-polar *phoP* mutation.

#### 2.1.4. Virulence of Wild-Type *P. multocida* and the Δ*phoP* Mutant

To determine the effect of the *phoP* mutation on bacterial virulence, the 50% lethal dose (LD_50_) of the parent strain and S413 (*P. multocida* Δ*phoP*) were measured in a duck animal model. Ducklings were inoculated with gradual doses of *P. multocida* via the intranasal and oral routes. The LD_50_ of S413 was 1.33 × 10^7^ CFU via the intranasal route, which was approximately 154-fold higher than the parent strain LD_50_ of 8.66 × 10^4^ (Table 1). The LD_50_ of S413 was 1.56 × 10^8^ CFU via the oral route, which was approximately 32-fold higher than the parent strain LD_50_ of 4.87 × 10^6^ (Table 1). These results indicate that the *phoP* deletion decreased the virulence of *P. multocida* 0818*.*

**Table 1:** Determination of the LD_50_ of *P. multocida* 0818 and the Δ*phoP* mutant.

Route	Strains	Infection Dose (CFU) and Survival							LD_50_
		10^3^	10^4^	10^5^	10^6^	10^7^	10^8^	10^9^	
Intranasal	*P. multocida* 0818	5/5	7/8	8/16	1/16	0/16	0/8	0/8	8.66 × 10^4^
	S413 (Δ*phoP*)	8/8	8/8	7/8	12/16	9/16	5/16	0/8	1.33 × 10^7^
Oral	*P. multocida* 0818	–	8/8	8/8	12/16	5/16	0/13	0/8	4.87 × 10^6^
	S413 (Δ*phoP*)	–	8/8	8/8	8/8	11/13	7/13	1/13	1.56 × 10^8^

–, not detected.

#### 2.1.5. Identification of Genes Regulated by *phoP* in *P. multocida*

Transcriptome sequencing was applied to search for genes regulated by *phoP* in *P. multocida*. Compared to the parent strain, 334 genes were differentially expressed in the S413 (Δ*phoP*) strain during the exponential growth stage, of which 161 genes were up-regulated and 173 genes were down-regulated (Supplementary Material, Table S1). The genes with a greater than four-fold difference in transcription between the parent strain and the Δ*phoP* mutant are listed in Table 2. Down-regulated genes with more than a four-fold difference were classified into several groups, including metabolism genes (*aspA*, *deaD*, PM0751, *rnc*, *resA*, *trmD* and *eptA*), membrane protein genes (PM0803, PM1164 and *tonB*), *infB* (translation), *nusA* (transcription), *dnaK* (RNA degradation) and PM1295 (unknown function). The genes that were up-regulated more than four-fold included *comF* (competence gene), PM0213 (membrane protein), stress reaction genes (*cspA* and *lexA*), PM1891 (mineral absorption), *def* (peptide deformylase), *impA* (type VI secretion protein), *rraA* (regulator of ribonuclease activity), *acpP* (acyl carrier protein, involved in fatty acid biosynthetic process) and PM1316 (unknown function). KEGG enrichment analysis showed that the regulated genes were significantly involved in five pathways, including bacterial secretion, RNA degradation, protein export, nitrogen metabolism and ribosome (Supplementary Material, Figure S1).

**Table 2:** Partial list of differentially expressed genes between the parent strain and the Δ*phoP* mutant.

GeneID ^a^	Locus	Description	Fold Change (log_2_)
Genes up-regulated in strain S413 (Δ*phoP*)			
1244903	*comF*	competence protein ComF	18.25
1243560	PM0213	membrane protein	11.79
1244002	*cspA*	cold-shock protein	5.98
1244528	*lexA*	repressor LexA	5.73
1245238	PM1891	hypothetical protein	5.39
1244663	PM1316	hypothetical protein	4.66
1244906	*def*	peptide deformylase, partial	4.66
1244239	*impA*	type VI secretion protein ImpA	4.29
1244515	*rraA*	ribonuclease activity regulator protein RraA	4.06
1245264	*acpP*	MULTISPECIES: acyl carrier protein	4.00
Genes down-regulated in strain S413 (Δ*phoP*)			
1244450	*aspA*	aspartate ammonia-lyase	9.92
1244459	*deaD*	RNA helicase	7.62
1244098	PM0751	DNA glycosylase	7.16
1244150	PM0803	TonB-dependent receptor	5.28
1243408	*rnc*	ribonuclease III	5.24
1243794	*resA*	*Pasteurella* ResA protein	5.03
1244644	*trmD*	tRNA (guanine-N1)-methyltransferase	4.96
1244642	PM1295	hypothetical protein	4.66
1244511	PM1164	membrane protein	4.66
1244107	*nusA*	peptidase M54	4.53
1244535	*tonB*	cell envelope protein TonB	4.47
1244106	*infB*	translation initiation factor IF-2	4.29
1244083	*dnaK*	molecular chaperone DnaK	4.14
1244389	*eptA*	sulfatase	4.11

^a^ NCBI accession number of the identified gene.

#### 2.1.6. Evaluation of the Immune Responses and Protection Rate Conferred by Δ*phoP* Mutant

**Figure 4:**
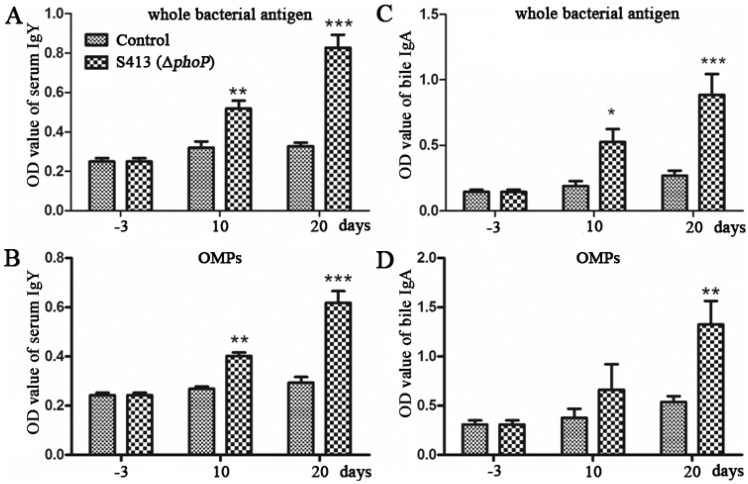
The antibody responses induced by the Δ*phoP* mutant in ducks. Ducks were immunized with 1 × 10^5^ CFU of Δ*phoP* mutant orally twice at an interval of 10 days. Then, the serum IgY responses specific to whole-bacterial antigen (**A**) and OMPs (**B**) and the bile IgA responses specific to whole-bacterial antigen (**C**) and OMPs (**D**) were measured by indirect ELISA. The data in B are expressed as the mean value ± SD and were analyzed with the significance levels set at 0.05 (*), 0.01 (**) and 0.001 (***).

To evaluate protective efficacy of strain S413 (Δ*phoP*), one-week-old ducks were immunized with 1 × 10^5^ CFU of S413 (Δ*phoP*) orally twice at an interval of 10 days. Then, ducks were challenged with approximately 200 × LD_50_ of the wild-type *P. multocida* 0818 at Day 10 after the second immunization (Table 3). The serum IgY and bile IgA responses specific to whole-bacterial antigen and OMPs were measured 3 days before immunization and 10 and 20 days post-immunization by indirect ELISA. No serum IgY or bile IgA responses specific to *P. multocida* antigens were present before immunization (Figure 4). In contrast, immunized ducks produced strong serum IgY and bile IgA antibodies against whole-bacterial antigen, both of which were significantly higher than that of the control ducks (Figure 4A,B). The serum IgY and bile IgA responses specific to *P. multocida* OMPs were significantly higher in the vaccinated group than in the control group (Figure 4C,D). In the immunized group, 54.5% of the ducks survived after challenge with 200 × LD_50_ of the wild-type *P. multocida* 0818, and all control ducks were dead within one week (Table 3), indicating that the Δ*phoP* mutant is a putative vaccine candidate that could provide moderate protection against *P. multocida* infection.

**Table 3:** Protection rate conferred by the Δ*phoP* mutant.

Group	Immunization	Challenge	Survival	Protection
Immune group	10^5^ CFU of S413 (Δ*phoP*)	10^9^ CFU of *P. multocida* 0818	6/11	54.5%
Control	PBS	10^9^ CFU of *P. multocida* 0818	0/8	0

### 2.2. Discussion

*P. multocida* is associated with a wide range of diseases in many species of animals. Determination of the roles of its global regulators will promote the understanding of bacterial survival and evasion in the host and will contribute to the development of vaccines. Recently, several global regulators have been identified in *Pasteurella*, such as Fis (nucleoid-associated protein) [25], Fur (ferric uptake regulation) [26,27] and FnrP [28,29], all of which are associated with bacteria virulence. PhoP and PhoQ constitute a two-component system that senses exterior signals and plays vital roles in bacterial virulence [30]. In this study, we found that the PM0214 gene (*phoP*2) of *P. multocida* performs *phoP* functions. Three main lines of evidence support this conclusion: (1) The *P. multocida* PM0214 gene has the ability to restore the resistance of a *S.* Typhimurium Δ*phoP* mutant to polymyxin B (Figure 1B); (2) The *P. multocida* PM0214 gene exhibited alkaline phosphatase activity (Figure 1A); (3) The PM0214 and PM0215 (NCBI Gene ID: 1243562) genes constitute an operon sharing the same promoter (Figure 2), and PM0215 (NCBI protein ID, WP_010906530.1) is an inner membrane protein sharing 45% similarity with PhoQ of *Salmonella,* which is the cognate sensor of PhoP. Therefore, the PM0214 gene of *P. multocida* is a *phoP* gene, which was interchangeable with *phoP* of *Salmonella*. In addition, the protein encoded by PM0432 (*phoP1*) shared only 27.23% identity to *Salmonella* PhoP and did not exhibit any alkaline phosphatase activity, which indicates of PhoP activity [31]. However, the protein shared approximately 60% identity to *E. coli* PhoB, which responds to phosphate starvation in *E. coli*. Hence, *phoP*1 might be the *phoB* gene of *P. multocida*. Considering the relationship between *phoP* mutation and virulence, we focused this study on the *phoP* gene of *P. multocida*.

The virulence of the S413 strain (Δ*phoP*) decreased 32- and 154-fold after oral and intranasal inoculation, respectively (Table 1). The reduction level of the *P*. *multocida* Δ*phoP* mutant was much lower than that of the *Salmonella* Δ*phoP* mutant, whose virulence was reduced by five orders of magnitude [32]. Similar results have been observed for the *phoP* mutation in *Yersinia pseudotuberculosis* [33], indicating that PhoP does not always have significant impacts on virulence. These results suggest that there are species-specific virulence aspects of *phoP* or that other genes in *P. multocida* may compensate for the adverse effects of a *phoP* deletion. The LD_50_ of *P. multocida* inoculated via the oral route was higher than that in the intranasal route, which was consistent with a previous study [34].

Global regulators are capable of regulating hundreds of genes essential for the virulence [30]. In this study, the *phoP* gene influenced the transcription of 334 genes, including 161 up-regulated genes and 173 down-regulated genes in *P. multocida*. Notably, three iron uptake genes, *tonB*, *exbD* and PM0803 (TonB-dependent receptor) were down-regulated in the absence of the *phoP* gene (Table S1). A previous report has demonstrated that *tonB* and *exbD* are necessary for the virulence of *P. multocida* cells and contribute equally to the infectious process, indicating that *tonB* and *exbD* down-regulation might partially contribute to the decreased virulence of the Δ*phoP*. The *phoP* gene was also involved in the down-regulation of some global regulators, such as *csrA* and *fis*, and the up-regulation of regulators such as *mraZ* (Table S1). CsrA, a post-transcriptional regulator, directly activates genes necessary for pedestal formation in enteropathogenic *E. coli* and for the type III secretion system in *P. aeruginosa* [35]. Fis is involved in the transcriptional regulation of genes encoding certain virulence factors in *P. multocida* [25], *Erwinia chrysanthemi* [36], *E. coli* [37] and *Salmonella* [38]. Overproduction of *E*. *coli* MraZ inhibits cell division and is lethal in rich medium [39]. Therefore, down-regulated CsrA and Fis and up-regulated MraZ in the *P*. *multocida* Δ*phoP* mutant may also partially account for its decreased virulence. Besides, *phoP* deletion down-regulated five membrane protein genes (Table S1). *Salmonella* PhoP/Q promotes the bacteria resistance to antimicrobial peptides by activating the synthesis of unique outer membrane proteins [40], indicating that these genes may be essential for *P*. *multocida* resistance to polymyxin B. In addtion, fourteen membrane protein genes, such as PM0213, an outer membrane protein with an 11.79-fold increased transcription level, were up-regulated in the Δ*phoP* mutant, which prompted us to investigate the function and immunogenicity of the fourteen proteins.

The *P*. *multocida* Δ*phoP* mutant stimulated strong serum IgY and bile IgA responses in ducks, indicating that Δ*phoP* mutant was a good vaccine candidate. Because the *P*. *multocida* Δ*phoP* mutant was not fully attenuated, low doses of this mutant were used to immunize ducks. Therefore, this mutant only provided approximately 55% protection against a challenge with 200 × LD_50_ of the *P. multocida* virulent strain (Table 3), which was a lower protective effect than was observed for the *Salmonella phoP* mutant [41,42] or the *Mycobacterium tuberculosis phoP* mutant [43]. Other mutations need to be introduced into the *phoP* mutant to achieve full attenuation for vaccine development. Our study provides a foundation to explore the mechanism of *phoP*-regulated genes and a platform for *P*. *multocida* attenuated vaccine development.

## 3. Experimental Section

### 3.1. Materials and Methods

#### 3.1.1. Bacterial Strains, Plasmids, Media and Growth Conditions

The bacterial strains and plasmids used in this study are listed in Table 4. The *P. multocida* strains were grown in BHI broth (BD Bioscience, San Jose, CA, USA). *S.* Typhimurium and *E. coli* were grown in LB broth. DAP was added to the LB broth at a final concentration of 50 μg/mL for growth of the *E. coli* and *S.* Typhimurium *asd* mutant strains. Solid media were prepared by the addition of 1.5% agar. When required, the media were supplemented with kanamycin (50 μg/mL) or chloramphenicol (25 μg/mL for *S.* Typhimurium and *E. coli*, 2.5 μg/mL for *P. multocida*). The plasmids were transformed into the bacterial strains by electroporation. Notably, *Salmonella* Δ*asd* strains and Asd^+^ plasmids were selected to identify the putative *phoP* gene of *P. multocida*. The *asd* gene encoding Asd is required for synthesis of DAP, an essential component for biosynthesis of cell walls of Gram^−^ bacteria. The *S.* Typhimurium Δ*asd* strain will undergo lysis in the absence of DAP unless exogenous Asd^+^ plasmid is retained in bacteria or DAP is provided [23,24]. Thus, the Asd^+^ plasmid carrying *phoP* gene can be stably maintained in *S.* Typhimurium Δ*asd* strains without antibiotic pressure post-transformation. 

**Table 4:** Bacterial strains and plasmids used in the study.

Strain or Plasmid	Description	Source
Strains		
S184	*S.* Typhimurium Δ*asd66*	Lab collection
S412	*S.* Typhimurium Δ*asd66* Δ*phoP85*	Lab collection
*P. multocida* 0818	Wild type *P. multocida* serotype A strain. Capsulated and virulent	Lab collection
S413	*P. multocida* 0818 Δ*phoP*::*kanR*	This work
χ7232	*E. coli* K-12, *endA1 hsdR17* (r_K_-, m_K_*+*) *glnV44 thi-1 recA1 gyrA relA1* Δ(*lacZYA-argF*)*U169 λpir deoR* (φ*80dlac* Δ(*lacZ*)*M15*)	[39]
χ7213	*E. coli* K-12, *thi-1 thr-1 leuB6 glnV44 fhuA21 lacY1 recA1 RP4-2-Tc*::Mu λ*pir* ∆*asdA4* ∆*zhf-2*::Tn*10*	[39]
Plasmids		
pQK664	Asd^+^ P_trc_ pSC101 origin replicationp15a, *asd*^+^, spec ^r^	Derived from pYA3337 [36]
pQK167	Insertion of *phoP1* into pQK664	This work
pQK168	Insertion of *phoP2* into pQK664	This work
pYA4278	pRE112 derivative, *sacB mobRP4* R6K *ori* Cm^+^	[38]
pQK171	pYA4278-Δ*phoP*	This work
pQK173	pYA4278-Δ*phoP*::*kanR*, for deletion of *phoP* in *P. multocida*	This work
pMC-Express	A broad host-range shuttle vector derived from pMIDG100, chloramphenicol ^r^	[40]
pQK180	Insertion of *phoP* into pMC-Express	This work

^r^ antibiotic resistance.

#### 3.1.2. Molecular and Genetic Procedures

DNA was amplified using the PrimeSTAR Max DNA polymerase (Takara Bio Inc., Shiga, Japan) or Taq DNA polymerase (Tiangen Biotech Co., Ltd., Beijing, China). The primers used in this study are listed in Table 5. When required, PCR products were purified using the Universal DNA Purification Kit (Tiangen Biotech) and commercially sequenced by BGI Tech (BGI Tech Solutions Co., Ltd., Shenzhen, China). Plasmids were restriction-digested and ligated according to the manufacturers’ instructions using enzymes obtained from NEB and TAKARA, respectively, and the plasmids were prepared using the TIANprep Mini Plasmid Kit (Tiangen Biotech).

**Table 5:** Primers used in the study.

Primers	Sequence 5’-3’
C*phoP1*-F	GCATGCCATGGAGGTACAAATGACCAAAAT
C*phoP1*-R	CGCGGATCCATACTTAATTGAAAAGGTTC
C*phoP2*-F	GCATGCCATGGCGGTCAAAAAAATGCGAAT
C*phoP2*-R	CGCGGATCCACGTGAAAATTAAACGAACC
C*phoP*-F	cgcGGATCCatgcgaattttattaatagaag
C*phoP*-R	ataagaatGCGGCCGCccgtaaacttgtttgcttaagc
D*phoP*-1F	TGCCAGTTTTCAATGGTGTC
D*phoP*-1R	CCTGCAGGGATGCGGCCGCTTTTTTGACCGCACTTTTTTC
D*phoP*-2F	GCGGCCGCATCCCTGCAGGGCTAGGAAAAAATGATGAAATG
D*phoP*-2R	ATTTTCCTTGATTGACTGGC
*Kan*-F	ATAAGAATGCGGCCGCTCAGTGGAACGAAAACTC
*Kan*-R	CCTGCAGGTTAGAAAAACTCATCGAGCATC
Primer 1	TTAATTGGCAATGGCTTACAG
Primer 2	TACCCTACACCATGCACAGT
Primer 3	TATTACACACGTTATAACCCG
Primer 4	CTGATTCAGGTGAAAATATTG
Primer 5	CATTTGTGCAACTTCAGTTTG
Primer 6	CAATATTTTCACCTGAATCAG
16sRNA-F	TAATACCGCGTATTCTCTGAGG
16sRNA-R	CCCTCCCTAAAGTACTCTAGAC

#### 3.1.3. Plasmid and Mutant Strain Construction

To clarify the function of the *phoP* gene of *P. multocida*, two pairs of primers, C*phoP*1-F/C*phoP*1-R and C*phoP*2-F/C*phoP*2-R, were used to amplify *phoP1* and *phoP2* from the *P. multocida* 0818 genome, respectively. The amplified DNA fragments and the Asd^+^ plasmid pQK664 derived from pYA3337 [44] were digested with NcoI and BamHI, and then the digested *phoP1* and *phoP2* PCR products were inserted into pQK664 to generate pQK167 and pQK168, respectively. 

A modification of the previously described single-crossover insertion mutagenesis method [45], which utilizes the λ pir-dependent plasmid pUA826, was applied for *phoP* deletion in *P. multocida*. To generate a non-polar mutation, a different suicide plasmid, T-vector pYA4278 [46] was used. In brief, the upstream and downstream fragments (approximately 450 bp) of the *phoP* gene were amplified from *P. multocida* 0818 genome with two pairs of primers, D*phoP*-1F/D*phoP*-1R and D*phoP*-2F/D*phoP*-2R (Figure 2A), respectively. The two fragments were then joined by PCR using the primers D*phoP*-1F and D*phoP*-2R. The terminal A was added at both ends to the resulting PCR product by using the Tailing-A Reaction Kit (Tiangen Biotech). The suicide plasmid T-vector pYA4278 was digested with AhdI to generate a linear T-vector, and the PCR product was ligated to this T-vector to generate the plasmid pQK171. Next, the *kanR* cassette amplified with the primers *kan*-F/*kan*-R was inserted into pQK171 between the NotI and SbfI sites to generate the plasmid pQK173. This plasmid was then mobilized from *E. coli* χ7213 [47] into *P. multocida* 0818 strain by conjugation, and the Δ*phoP* mutant termed S413 was selected on BHI agar containing 50 μg/mL kanamycin. Finally, the selected mutant was confirmed by PCR using primers 1, 2, 3, 4, 5 and 6, which flanked the genomic sequence and the *kanR* cassette. As a positive control, the 16S ribosomal RNA gene was amplified with the primers 16sRNA-F/16sRNA-R, which were designed according to the NCBI sequence JX869945.1.

To complement the *phoP* mutant in *P. multocida*, the *phoP* gene sequence was amplified from *P. multocida* 0818 genomic DNA using the primers C*phoP*-F/C*phoP*-R containing NotI and BamHI sites. The amplified fragment was digested and cloned into NotI-BamHI-digested pMC-Express (kindly provided by Paul R Langford from Imperial College London) [48], generating pQK180. The plasmids pQK167 and pQK168 were separately transformed into S412 (*S.* Typhimurium Δ*asd* Δ*phoP*), and pQK180 was transformed into S413 (*P. multocida* Δ*phoP*), generating the corresponding complementary strains S412 (pQK167), S412 (pQK168) and S413 (pQK180).

#### 3.1.4. Phosphatase Activity and Polymyxin B Resistance Assays

To detect phosphatase activity, X-P plates were obtained by addition of 5-bromo-4-chloro-3-indolyl-phosphate (BCIP) (Sigma-Aldrich, St. Louis, MO, USA) to LB agar at a final concentration of 50 μg/mL [31]. Four *S.* Typhimurium strains, S184 (Δ*asd*) harboring the control plasmid pQK664, S412 (Δ*asd* Δ*phoP*) harboring pQK664, S412 harboring pQK167 and S412 harboring pQK168, were grow at 37 °C on X-P plates for 18 h, and the colony color was observed.

To detect the resistance to polymyxin B, bacteria were grown to an OD_600_ of 0.8 in LB broth (*S.* Typhimurium) or BHI broth (*P. multocida*), harvested, and washed in PBS. The cells were diluted to 1 × 10^6^ CFU and were cultured at 37 °C in culture media supplemented with or without polymyxin B (0.1 μg/mL for *S.* Typhimurium and 0.5 μg/mL for *P. multocida*) for 1 h [49]. Then, the cells were 10-fold serially diluted and 100 μL of the diluted suspension was spread on LB agar or BHI agar to determine the number of bacteria. Percent survival was calculated as the mean CFU of the polymyxin B-treated group divided by the mean CFU of the untreated group.

#### 3.1.5. Phenotype Determinations

The *P. multocida* 0818 and S413 (*P. multocida* Δ*phoP*) stains were grown in BHI broth, and the OD_600_ values were recorded every 2 h over a period of 14 h to generate the growth curves.

LPS of *P. multocida* was prepared as previously described [50]. *P. multocida* was grown in BHI broth overnight, harvested and washed three times using distilled water. The cells were re-suspended in 150 µL of sample buffer (0.5 M Tris–HCl pH 6.8, 2% SDS, 0.05% ethyl alcohol, 10% glycerol, 5% β-mercaptoethanol), boiled for 10 min, and centrifuged at 10,000× *g* for 10 min. Ten microliters of the supernatant was mixed with 90 µL of loading buffer and 1 µL of proteinase K (20 mg/mL, Sigma-Aldrich), followed by incubation for 1 h at room temperature. Next, 15 µL of the mixture was subjected to 12.5% SDS-PAGE, and the SDS-PAGE gels were visualized using a standard silver-staining protocol [51]. 

The OMPs of *P. multocida* were extracted as previously described [52]. *P. multocida* was harvested after growth to an OD_600_ of 0.8, and the pellet was re-suspended in 2 mL of HEPES buffer (10 mM, pH 7.4) on ice. The cells were disrupted by sonication (six bursts, 10 s each), and the unbroken cells were removed by centrifugation (15,600× *g*, 2 min, 4 °C). The supernatant containing the OMPs was transferred into a new tube and centrifuged again (15,600× *g*, 30 min, 4 °C). The membrane pellet was re-suspended in 0.2 mL of HEPES buffer. To solubilize the cytoplasmic membrane, 0.2 mL of 2% sarcosyl was added and incubated at room temperature for 30 min with constant shaking. After centrifugation (15,600× *g*, 30 min, 4 °C), the pellet containing the OMPs was washed once with 0.5 mL of HEPES buffer and re-suspended in 50 µL of HEPES buffer. The OMP concentration was measured using a BCA Protein Assay Kit (Thermo Scientific, Rockford, IL, USA). Next, the protein samples were prepared in sample buffer (50 mM Tris, 5% β-mercaptoethanol, 20% glycerol, 0.005% bromophenol blue, 4% SDS) and boiled at 95 °C for 5 min. The samples were analyzed by 12.5% SDS-PAGE and were subjected to staining with Coomassie Brilliant Blue R-250 (Sigma-Aldrich) for protein band detection.

#### 3.1.6. Determination of LD_50_ in Ducks

All experiments with animals in this study were performed in strict accordance with the recommendations in the Guide for the Care and Use of Laboratory Animals of the Ministry of Science and Technology of China. All animal procedures were approved by the Animal Ethics Committee of the Sichuan Agricultural University (Approval No. 2015-015, Approval date: January 2015).

One-day-old Sheldrake ducks were purchased from a hatchery and acclimated for 7 days after arrival before starting the experiments. For determination of the 50% lethal dose (LD_50_), the *P. multocida* strains were grown statically overnight at 37 °C in BHI broth, diluted 1:100 in fresh medium and grown with aeration (180 rpm) at 37 °C. When the cultures reached an OD_600_ of 0.8–0.9, they were harvested at room temperature at 4000 rpm, washed in PBS, and suspended in PBS. Groups of 1-week-old ducks (5–16 ducks per group) were infected orally with 500 μL of PBS containing various doses of *P. multocida* 0818 or S413 (Δ*phoP*) ranging from 1 × 10^4^ to 1 × 10^9^ CFU. Meanwhile, groups of 2-week-old ducks were infected intranasally with 100 μL of PBS containing various doses of *P. multocida* 0818 or S413 (Δ*phoP*) strains, ranging from 1 × 10^3^ to 1 × 10^9^ CFU. The animals were observed for 2 weeks post-infection, and deaths were recorded daily. The LD_50_ was calculated with the method of Reed and Muench.

#### 3.1.7. Transcriptome Sequencing

RNA sequencing was performed on an Illumina HiSeq2500/MiSeq instrument (Illumina, San Diego, CA, USA) by Shanghai Majorbio Bio-pharm Biotechnology Co., Ltd. (Shanghai, China). For preparation of the samples, the *P. multocida* 0818 and S413 (Δ*phoP*) strains were cultured in BHI broth to an OD_600_ of 0.8, and the total RNA was isolated from each sample with Trizol Reagent (Invitrogen, Carlsbad, CA, USA). The ribosomal RNA was removed from RNA samples before construction of the cDNA library and Illumina deep sequencing.

For sequence analysis, we chose the published sequence of *P. multocida* PM70 as the reference genome sequence [53], and the clean reads from each sample were mapped to isogenes using Bowtie 2 software [54]. The number of mapped reads per kilobase of exon per million mapped reads (RPKM) was used as the transcription activity of each gene. The differentially expressed genes were analyzed using RSEM and edgeR software [55,56]. The RPKM of genes showing a ≥2-fold intensity change between *P. multocida* 0818 and S413 with a false discovery rate (FDR) of <0.5 were considered differentially expressed.

#### 3.1.8. Immunization and Challenge

Seventeen 1-week-old commercial Sheldrake ducks were inoculated with 500 μL of PBS containing 1 × 10^5^ CFU of S413 orally on Day 0 and were boosted on Day 10 with the same dose of the same strain. Another 14 ducks were inoculated with 500 μL of PBS as unimmunized controls. Blood and bile were collected from three randomly selected ducks in both groups on Day –3, Day 10, and Day 20. Blood was allowed to coagulate at 37 °C for 2 h. Following centrifugation, the serum was removed and stored at −80 °C. For the challenge experiment, the remaining immunized (11 ducks) and control ducks (8 ducks) were challenged orally with 500 μL of PBS containing 1 × 10^9^ of *P. multocida* strain 0818. The animals were observed for 2 weeks post-challenge, and deaths were recorded daily. Necropsy and bacterial isolation were carried out routinely after death.

#### 3.1.9. Enzyme-Linked Immunosorbent Assay (ELISA)

The serum IgY and bile IgA responses were measured by indirect ELISA as previously described [34]. *P. multocida* strain 0818 was inactivated by heating at 80 °C for 10 min in a water bath, and the nonviable bacteria were washed in saline and suspended to approximately 1 × 10^11^ CFU/mL (as determined by viable counts prior to boiling). A 1:100 dilution of the whole-cell antigen or 0.25 μg/mL purified OMPs were prepared in carbonate-bicarbonate buffer (pH 9.6), and 100 μL of antigen was added to each well of a 96-well ELISA microtiter plate (Nunc-immuno MaxiSorp plate, Nunc, Roskilde, Denmark). The plate was incubated at 4 °C overnight. After washing and blocking, the plate was incubated with 1:160 diluted serum or 1:40 diluted bile for 1 h at 37 °C. Then, 100 μL of 1:5000 diluted alkaline phosphatase (AP)-labeled mouse anti-duck IgY or IgA (AbD Serotec, Puchheim, Germany) was added to the wells, and the plate was incubated at 37 °C for 1 h followed by four washes. AP solution (Sigma-Aldrich) was added to the wells for 20 min. The reaction was stopped by the addition of 100 μL of 0.2 M NaOH. The plate was read at 415 nm by an ELISA reader (Bio-Rad Laboratories, Richmond, CA, USA).

#### 3.1.10. Statistical Analyses

Statistical analysis was performed using the GraphPad Prism 5 software package (Graph Software, San Diego, CA, USA). Data are expressed as the means ± SD and were analyzed using Student’s *t*-test at significance levels of 0.05, 0.01 and 0.001. The animal experiments were repeated at least twice, and the *in vitro* experiments were performed independently three times in triplicate. The representative results are presented in the study.

## 4. Conclusions

In this study, we identified the *phoP* gene in *P*. *multocida* and constructed and characterized a *P*. *multocida* Δ*phoP* mutant. Compared to the parent strain, this mutant is more sensitive to polymyxin B, exhibits lower virulence in ducks, and provides moderate immune protection against challenge with virulent *P*. *multocida* strains. 

## Supplementary Materials

Supplementary materials can be found at http://www.mdpi.com/1422-0067/17/1/12/s1.
