# Supplementary material for: Identification of the Avian Pasteurella multocida phoP Gene and Evaluation of the Effects of phoP Deletion on Virulence and Immunogenicity
Source: Int J Mol Sci. 2015 Dec 23;17(1):12. doi: 10.3390/ijms17010012 (PMC4730259; doi:10.3390/ijms17010012)
Supplement: Supplementary file 1 [file ijms-17-00012-s001.pdf]

Supplementary Materials: Identification of the Avian *Pasteurella multocida phoP* Gene and Evaluation of the Effects of *phoP* Deletion on Virulence and Immunogenicity

Kangpeng Xiao, Qing Liu, Xueyan Liu, Yunlong Hu, Xinxin Zhao and Qingke Kong

Table S1. The total list of differentially expressed genes between the parent strain and the Δ*phoP* mutant.

Gene ID [a]	Locus	Description	Fold Change (log2)
		**Genes up-regulated in S413 (Δ*phoP*) strain**	
1244903	*comF*	competence protein ComF	18.25
1243560	PM0213	membrane protein	11.79
1244002	*cspA*	cold-shock protein	5.98
1244528	*lexA*	LexA repressor	5.73
1245238	PM1891	hypothetical protein	5.39
1244663	PM1316	hypothetical protein	4.66
1244906	*def*	peptide deformylase, partial	4.66
1244239	*impA*	type VI secretion protein ImpA	4.29
1244515	*rraA*	ribonuclease activity regulator protein RraA	4.06
1245264	*acpP*	MULTISPECIES: acyl carrier protein	4.00
1244599	PM1252	hypothetical protein	3.97
1244152	PM0805	hypothetical protein	3.94
1244825	PM1478	hypothetical protein	3.81
1244475	PM1128	dithiol-disulfide isomerase	3.78
1244121	*hyaE*	protein HyaE	3.76
1243738	PM0391	hypothetical protein PMCN03_0396	3.56
1245185	*pnuC*	nicotinamide riboside transporter pnuC	3.46
1245181	PM1834	hypothetical protein	3.46
1243560	*arsC*	arsenate reductase	3.46
1243784	*rrmJ*	23S rRNA methyltransferase	3.48
1244911	*mscL*	large-conductance mechanosensitive channel	3.41
1244568	PM1221	hypothetical protein	3.39
1244417	PM1070	hypothetical protein	3.36
1244547	*hisB*	imidazoleglycerol-phosphate dehydratase	3.36
1243853	PM0506	hypothetical protein	3.25
1243815	*htpX*	heat shock protein HtpX	3.25
1243559	PM0212	hypothetical protein	3.25
1244915	PM1568	hypothetical protein	3.25
1243763	*pgi*	glucose-6-phosphate isomerase	3.23
1244716	*gpt*	xanthine phosphoribosyltransferase	3.16
1244437	PM1090	membrane protein	3.16
1243552	PM0205	hypothetical protein	3.14
1244380	*corC*	magnesium transporter	3.14
1243679	*recN*	recombinase	3.10
1244613	*thiZ*	phosphonate ABC transporter ATP-binding protein	3.10
1244033	PM0686	SAM-dependent methyltransferase	3.07
1244843	PM1496	sulfurtransferase	2.99
1244912	PM1565	regulator	2.99
1244691	*deoR*	transcriptional regulator	2.99
1244503	PM1156	hypothetical protein	2.93
1244410	*mtlR*	mannitol repressor protein	2.89
1244633	*uspA*	universal stress protein A	2.89
1244576	*comA*	competence protein ComA	2.89

Table S1. *Cont.*

Gene ID [a]	Locus	Description	Fold Change (log2)
1245307	*leuC*	3-isopropylmalate dehydratase large subunit	2.87
1244767	*tnaA*	L-cysteine desulfhydrase	2.87
1243618	PM0271	hypothetical protein	2.87
1245043	*plsY*	glycerol-3-phosphate acyltransferase	2.85
1243875	*glnS*	glutaminyl-tRNA synthetase	2.85
1244823	*gyrB*	DNA gyrase subunit B	2.85
1244830	PM1483	cell division protein ZapB	2.83
1245233	*smpA*	membrane protein SmpA	2.83
1244077	*nusB*	transcription antitermination protein NusB	2.81
1245308	*leuB*	3-isopropylmalate dehydrogenase	2.79
1245146	*mobA*	molybdopterin-guanine dinucleotide biosynthesis protein MobA	2.79
1243883	PM0536	hypothetical protein	2.79
1243615	*proQ*	osmoprotectant transporter ProQ	2.73
1244518	PM1171	hypothetical protein	2.73
1244699	*tusC*	sulfur relay protein TusC	2.71
1244405	PM1058	hypothetical protein	2.69
1244970	PM1623	membrane protein	2.68
1244040	*folE*	GTP cyclohydrolase I	2.66
1244378	*infA*	MULTISPECIES: translation initiation factor IF-1	2.62
1245100	*mgtC*	membrane protein	2.62
1244700	*tusB*	hypothetical protein	2.57
1243738	PM0391	hypothetical protein	2.57
1244813	*yjgF*	endoribonuclease L-PSP	2.55
1244561	*leuS*	leucyl-tRNA synthetase	2.53
1244015	*fnr*	fumarate/nitrate reduction transcriptional regulator	2.51
1243979	PM0632	hypothetical protein	2.51
1243772	PM0425	heme ABC transporter ATP-binding protein	2.51
1243504	PM0157	hypothetical protein	2.50
1244011	*tyrA*	prephenate dehydrogenase	2.48
1245182	PM1835	aminotransferase	2.48
1244281	PM0934	hypothetical protein	2.48
1245136	PM1789	RNA polymerase sigma factor AlgU	2.48
1245072	*ubiH*	2-octaprenyl-3-methyl-6-methoxy-1,4-benzoquinol hydroxylase	2.46
1244860	PM1513	rRNA methyltransferase	2.45
1245315	*srlD*	sorbitol-6-phosphate dehydrogenase	2.45
1243455	PM0108	hypothetical protein	2.45
1244313	*pal*	membrane protein	2.43
1244036	PM0689	membrane protein	2.43
1243681	*grpE*	molecular chaperone GrpE	2.41
1243358	*menB*	dihydroxynaphthoic acid synthetase	2.41
1244351	PM1004	hypothetical protein	2.41
1245110	*sugC*	sugar ABC transporter ATP-binding protein	2.38
1243501	*rbsC*	ribose ABC transporter permease	2.38
1244574	PM1227	Competence protein, Chromosome segregation ATPase	2.38
1244855	PM1508	hypothetical protein	2.36
1244826	PM1479	beta-lactamase	2.35
1244309	*ppdC*	hypothetical protein	2.35
1244035	PM0688	membrane protein	2.35
1244201	PM0854	hypothetical protein	2.35
1243743	*acyP*	acylphosphatase	2.33
1243790	*minC*	septum site-determining protein MinC	2.31
1243761	*pglA*	glycosyl transferase family A	2.31
1244873	PM1526	C4-dicarboxylate ABC transporter	2.31

Table S1. *Cont.*

Gene ID [a]	Locus	Description	Fold Change (log2)
1243622	*dctP*	C4-dicarboxylate ABC transporter	2.30
1244476	*arfA*	hypothetical protein	2.30
1244214	*sanA*	membrane protein;Pasteurella protein SanA	2.28
1244868	*ftsX*	cell division protein FtsX	2.27
1243714	PM0367	membrane protein	2.27
1244706	PM1359	gluconate permease	2.27
1244047	*ubiX*	3-octaprenyl-4-hydroxybenzoate carboxy-lyase	2.27
1243980	*rluA*	RNA pseudouridine synthase	2.23
1245329	*rbbA*	multidrug ABC transporter ATP-binding protein	2.23
1244977	*crcB*	chromosome condensation protein CcrB	2.23
1244392	*ybeY*	metal-binding heat shock protein	2.23
1244399	*bcp*	thioredoxin-dependent thiol peroxidase	2.22
1244219	*hns*	DNA-binding protein	2.22
1244429	PM1082	hypothetical protein	2.20
1243429	*nudH*	RNA pyrophosphohydrolase	2.20
1244104	*rbfA*	ribosome-binding factor A	2.20
1244867	*ftsE*	cell division protein FtsE	2.20
1244607	*thiE*	thiamine-phosphate pyrophosphorylase	2.20
1244060	*yhbY*	RNA-binding protein	2.19
1244203	*pgsA*	CDP-diacylglycerol--glycerol-3-phosphate	2.19
1244575	*comB*	ComB	2.19
1244931	PM1584	RNA polymerase factor sigma-32	2.17
1243480	*mraZ*	cell division protein MraZ	2.17
1245317	*srlA*	PTS sorbitol transporter subunit IIB	2.17
1244312	*ppdA*	hypothetical protein	2.14
1245218	*eno*	enolase	2.13
1244111	*sgaB*	PTS ascorbate transporter subunit IIBC	2.13
1243432	*hofB*	protein transporter HofB	2.13
1244430	PM1083	hypothetical protein	2.13
1243367	*purN*	phosphoribosylglycinamide formyltransferase	2.13
1244453	*groES*	MULTISPECIES: co-chaperonin GroES	2.11
1245109	PM1762	sugar ABC transporter substrate-binding protein	2.11
1244268	*rpoZ*	DNA-directed RNA polymerase subunit omega	2.10
1244659	PM1312	hypothetical protein	2.10
1245223	*rph*	ribonuclease PH	2.10
1243448	PM0101	membrane protein	2.10
1245231	PM1884	hypothetical protein	2.10
1245168	PM1821	membrane protein	2.08
1244990	PM1643	C4-dicarboxylate ABC transporter substrate-binding protein	2.08
1245101	*dmsA*	dimethyl sulfoxide reductase subunit A	2.08
1244767	*ispF*	2-C-methyl-D-erythritol 2,4-cyclodiphosphate synthase	2.08
1244146	*yciS*	membrane protein	2.08
1245041	*cysZ*	cysteine biosynthesis protein CysZ	2.08
1244988	PM1641	metal-dependent hydrolase	2.08
1244874	PM1527	C4-dicarboxylate ABC transporter permease	2.07
1244822	PM1475	transporter	2.07
1244600	PM1253	C4-dicarboxylate ABC transporter permease	2.07
1243869	*sspA*	starvation protein A	2.07
1244797	*greB*	transcription elongation factor GreB	2.06
1244243	*crr*	PTS glucose transporter subunit IIA	2.06
1243700	PM0353	flavodoxin,Pasteurella flavodoxin FldA	2.06
1244927	PM1580	C4-dicarboxylate ABC transporter permease	2.06
1243663	*trmJ*	rRNA methyltransferase	2.04

Table S1. *Cont.*

Gene ID [a]	Locus	Description	Fold Change (log2)
1244851	PM1504	dimethyladenosine transferase	2.04
1245075	*metN*	methionine ABC transporter ATP-binding protein	2.03
1244671	*bolA*	BolA	2.03
1245145	PM1798	hypothetical protein	2.03
1244200	PM0853	hypothetical protein	2.03
1244158	*tnaA*	L-cysteine desulfhydrase	2.03
1243987	PM0640	alpha-amylase	2.03
1244834	*atpI*	F0F1 ATP synthase subunit I	2.03
1244156	*virK*	membrane protein	2.01
1244717	*proS*	prolyl-tRNA synthetase	2.00
1245076	*metI*	methionine ABC transporter permease	2.00
1244327	PM0980	hypothetical protein	2.00
		**Genes down-regulated in S413 (Δ*phoP*) strain**	
1244450	*aspA*	aspartate ammonia-lyase	9.92
1244459	*deaD*	RNA helicase	7.62
1244098	PM0751	DNA glycosylase	7.16
1244150	PM0803	TonB-dependent receptor	5.28
1243408	*rnc*	ribonuclease III	5.24
1243794	*resA*	ResA protein,Pasteurella ResA protein	5.03
1244644	*trmD*	tRNA (guanine-N1)-methyltransferase	4.96
1244642	PM1295	hypothetical protein	4.66
1244511	PM1164	membrane protein	4.66
1244107	*nusA*	peptidase M54	4.53
1244535	*tonB*	cell envelope protein TonB	4.47
1244106	*infB*	translation initiation factor IF-2	4.29
1244083	*dnaK*	molecular chaperone DnaK	4.14
1244389	*eptA*	sulfatase	4.11
1245047	PM1700	HAD family hydrolase	3.94
1245086	*rplJ*	50S ribosomal protein L10	3.94
1244088	PM0741	ligand-gated channel protein	3.71
1244635	*csrA*	carbon storage regulator	3.68
1244681	*apbE*	thiamine biosynthesis lipoprotein ApbE	3.68
1243799	PM0452	iron transporter	3.56
1244433	*fis*	MULTISPECIES: Fis family transcriptional regulator	3.56
1243608	*potD*	putrescine/spermidine ABC transporter substrate-binding protein	3.48
1244510	*rnpA*	ribonuclease P	3.46
1245332	*tsf*	endo-1,4-D-glucanase	3.41
1243798	PM0451	membrane protein	3.39
1243730	*rnfG*	electron transporter RnfG	3.27
1244626	*lsrG*	autoinducer-2 (AI-2) modifying protein LsrG	3.20
1244261	*dppD*	peptide ABC transporter ATP-binding protein	3.18
1244427	*fepC*	ferrichrome ABC transporter ATP-binding protein	3.16
1244338	*rne*	ribonuclease E	3.14
1243962	*topA*	topoisomerase I	3.10
1245351	*glnB*	nitrogen regulatory protein P-II	3.10
1244078	*ribH*	6,7-dimethyl-8-ribityllumazine synthase	3.07
1244141	*metC*	cystathionine beta-lyase	3.07
1243917	*tesB*	acyl-CoA thioesterase	3.07
1244244	*ptsI*	phosphoenolpyruvate-protein phosphotransferase	2.99
1244742	*secY*	preprotein translocase subunit SecY	2.91
1244942	*napG*	quinol dehydrogenase	2.89
1244052	*pta*	phosphate acetyltransferase	2.89
1243858	PM0511	glycosyltransferase	2.89

Table S1. *Cont.*

Gene ID [a]	Locus	Description	Fold Change (log2)
1245011	*ispH*	4-hydroxy-3-methylbut-2-enyl diphosphate reductase	2.89
1244325	*ruvC*	Holliday junction resolvase	2.89
23340310	*nrfF*	protein NrfF	2.85
1244442	PM1095	hypothetical protein	2.85
1245140	*torA*	trimethylamine N-oxide reductase I catalytic subunit	2.83
1243555	*secG*	preprotein translocase subunit SecG	2.83
1244840	*atpG*	F0F1 ATP synthase subunit gamma	2.83
1243610	*potB*	putrescine/spermidine ABC transporter permease	2.81
1244315	*tolA*	cell envelope biogenesis protein TolA	2.77
1244460	*nlpI*	hypothetical protein	2.77
1243970	*moaD*	molybdopterin synthase small subunit	2.77
1245085	*rplL*	50S ribosomal protein L7/L12	2.75
1244838	*atpH*	F0F1 ATP synthase subunit delta	2.75
1243665	*iscS*	cysteine desulfurase	2.75
1245060	PM1713	hypothetical protein	2.73
1243873	*cutC*	copper homeostasis protein CutC	2.73
1243574	*secD*	preprotein translocase subunit SecD	2.71
1244304	*fbpC*	sugar ABC transporter	2.71
1243904	PM0557	hypothetical protein	2.71
1243635	*lldD*	L-lactate dehydrogenase	2.68
1244254	*hflX*	GTPase HflX	2.68
1243949	*infC*	translation initiation factor IF-3	2.68
1243541	PM0194	5'-methylthioadenosine/*S*-adenosylhomocysteine nucleosidase	2.68
1243355	*ccmD*	hemagglutination activity protein	2.68
1243360	*ccmH*	hypothetical protein	2.66
1244085	*metC*	hypothetical protein	2.66
1245084	*rpoB*	DNA-directed RNA polymerase subunit beta	2.66
1244841	*atpD*	F0F1 ATP synthase subunit beta	2.64
1244434	*dusB*	tRNA-dihydrouridine synthase	2.60
1245220	PM1873	branched-chain amino acid ABC transporter substrate-binding protein	2.58
1244732	*ribB*	3,4-dihydroxy-2-butanone 4-phosphate synthase	2.58
1245200	PM1853	hypothetical protein	2.57
1244682	PM1335	hypothetical protein	2.57
1243409	*lepB*	signal peptidase I	2.55
1244651	*coaD*	phosphopantetheine adenylyltransferase	2.55
1244224	*uup*	heme ABC transporter ATPase	2.51
1245018	PM1671	3-phosphoglycerate dehydrogenase	2.51
1244319	PM0972	hypothetical protein	2.50
1245257	*oppA, mppA*	peptide ABC transporter substrate-binding protein	2.50
1244897	*devB*	6-phosphogluconolactonase	2.48
1244703	*fusA*	elongation factor G	2.48
1245152	PM1805	hypothetical protein	2.46
1243580	PM0233	protease	2.46
1244939	*napF*	ferredoxin	2.46
1244894	*hslO*	molecular chaperone Hsp33	2.46
1245261	*fabH*	3-oxoacyl-ACP synthase	2.45
1243527	*murA*	UDP-N-acetylglucosamine 1-carboxyvinyltransferase	2.43
1243706	*aroC*	chorismate synthase	2.41
1245246	*psd*	phosphatidylserine decarboxylase	2.41
1243363	PM0016	hypothetical protein	2.41
1243707	*mepA*	penicillin-insensitive murein endopeptidase	2.39

Table S1. *Cont.*

Gene ID [a]	Locus	Description	Fold Change (log2)
1244007	*cysB*	CysB family transcriptional regulator	2.38
1245046	PM1699	hypothetical protein	2.35
1244205	*kdsB*	3-deoxy-manno-octulosonate cytidylyltransferase	2.35
1244839	*atpA*	F0F1 ATP synthase subunit alpha	2.35
1244084	PM0737	transcriptional regulator	2.35
1244739	*rpsK*	MULTISPECIES: 30S ribosomal protein S11	2.33
1243668	*hscB*	CoA-transferase	2.33
1245276	*lipB*	lipoate-protein ligase B	2.33
1244416	*fadL*	membrane protein	2.33
1243636	*tilS*	tRNA(Ile)-lysidine synthetase	2.33
1244087	*dnaJ*	molecular chaperone DnaJ	2.31
1245201	PM1854	amino acid dehydrogenase	2.30
1245358	*hisS*	histidyl-tRNA synthetase	2.30
1244367	*nudF*	ADP-ribose pyrophosphatase	2.30
1244480	PM1133	hypothetical protein	2.30
1244675	*nqrA*	Na(+)-translocating NADH-quinone reductase subunit A	2.28
1244895	*cysQ*	2′, 3′-cyclic nucleotide 2′-phosphodiesterase	2.28
1244536	PM1189	hypothetical protein	2.28
1245141	*torD*	molecular chaperone TorD	2.28
1244677	*nqrC*	Na(+)-translocating NADH-quinone reductase subunit C	2.27
1245274	PM1927	D-alanyl-D-alanine carboxypeptidase	2.27
1244987	*tpiA*	triosephosphate isomerase	2.27
1243829	PM0482	hypothetical protein	2.25
1243905	*kdsA*	2-dehydro-3-deoxyphosphooctonate aldolase	2.25
1244368	PM1021	peptidase M15	2.25
1244275	*mltA*	murein transglycosylase	2.25
1243383	*sdaA*	serine dehydratase	2.25
1245083	*rpoC*	DNA-directed RNA polymerase subunit beta'	2.23
1243956	*mukB*	cell division protein MukB	2.23
1244066	PM0719	ribonucleotide-diphosphate reductase subunit beta	2.22
1245227	*dusC*	tRNA-dihydrouridine synthase C	2.22
1243921	*ybbN*	hypothetical protein	2.22
1243358	*dsbE*	thiol:disulfide interchange protein	2.22
1245275	PM1928	hypothetical protein	2.20
1245142	*fruA*	PTS fructose transporter subunit IIB	2.20
1243728	PM0381	endonuclease IV	2.20
1244008	*rluB*	ribosomal large subunit pseudouridine synthase B	2.20
1244439	PM1092	acetyl-CoA carboxylase biotin carboxyl carrier protein	2.19
1244135	PM0788	acyl-CoA esterase	2.19
1245241	PM1894	hypothetical protein	2.17
1244935	PM1588	RNA polymerase subunit sigma-32	2.17
1244664	*trmB*	tRNA (guanine-N(7)-)-methyltransferase	2.17
1243864	*recD*	exodeoxyribonuclease V	2.16
1243464	*hflK*	membrane protease HflK	2.16
1243573	*secF*	preprotein translocase subunit SecF	2.14
1243928	*trpD*	anthranilate phosphoribosyltransferase	2.14
1245356	*rodZ*	hypothetical protein	2.14
1244837	*atpF*	F0F1 ATP synthase subunit B	2.14
1244963	PM1616	membrane protein	2.13
1244737	*rpoA*	DNA-directed RNA polymerase subunit alpha	2.13
1243817	PM0470	multidrug ABC transporter ATP-binding protein	2.13
1243532	PM0185	6-carboxy-5,6,7,8-tetrahydropterin synthase	2.13
1244032	*gloB*	hydroxyacylglutathione hydrolase	2.13

Table S1. *Cont.*

Gene ID [a]	Locus	Description	Fold Change (log2)
1244328	*nudB*	dihydroneopterin triphosphate pyrophosphatase	2.11
1245262	*fabD*	malonyl CoA-ACP transacylase	2.11
1243903	*hemK*	N5-glutamine *S*-adenosyl-L-methionine-dependent methyltransferase	2.10
1244204	*uvrC*	excinuclease ABC subunit C	2.10
1244252	*miaA*	tRNA delta(2)-isopentenylpyrophosphate transferase	2.10
1243940	*thrS*	threonyl-tRNA synthetase	2.10
1245277	*lipA*	lipoyl synthase	2.08
1243955	*mukE*	condesin subunit E	2.08
1243823	*queF*	7-cyano-7-deazaguanine reductase	2.08
1244479	PM1132	transporter	2.07
1244783	*glpE*	thiosulfate sulfurtransferase	2.07
1245263	*fabG*	3-ketoacyl-ACP reductase	2.07
1245339	PM1992	hypothetical protein	2.07
1244729	*speA*	arginine decarboxylase	2.07
1245357	*ispG*	4-hydroxy-3-methylbut-2-en-1-yl diphosphate synthase	2.07
1243702	PM0355	esterase	2.06
1245134	*rseB*	sigma-E factor regulatory protein RseB	2.06
1243357	*ccmF*	heme lyase subunit CcmF	2.06
1243507	PM0160	allantoate amidohydrolase	2.06
1245161	*hemX*	hypothetical protein	2.06
1244653	*waaE*	glycosyltransferase	2.04
1245355	*pilF*	hypothetical protein	2.04
1244079	*tyrP*	tyrosine transporter	2.04
1244534	*exbD*	biopolymer transporter ExbD	2.03
1243594	*cca*	CCA-adding protein	2.03
1244680	*nqrF*	Na(+)-translocating NADH-quinone reductase subunit F	2.03
1244945	*napC*	cytochrome C	2.01
1243766	PM0419	hydrolase	2.01
1243519	*lptA*	sugar ABC transporter substrate-binding protein	2.01

[a]: NCBI accession number of the identified gene.

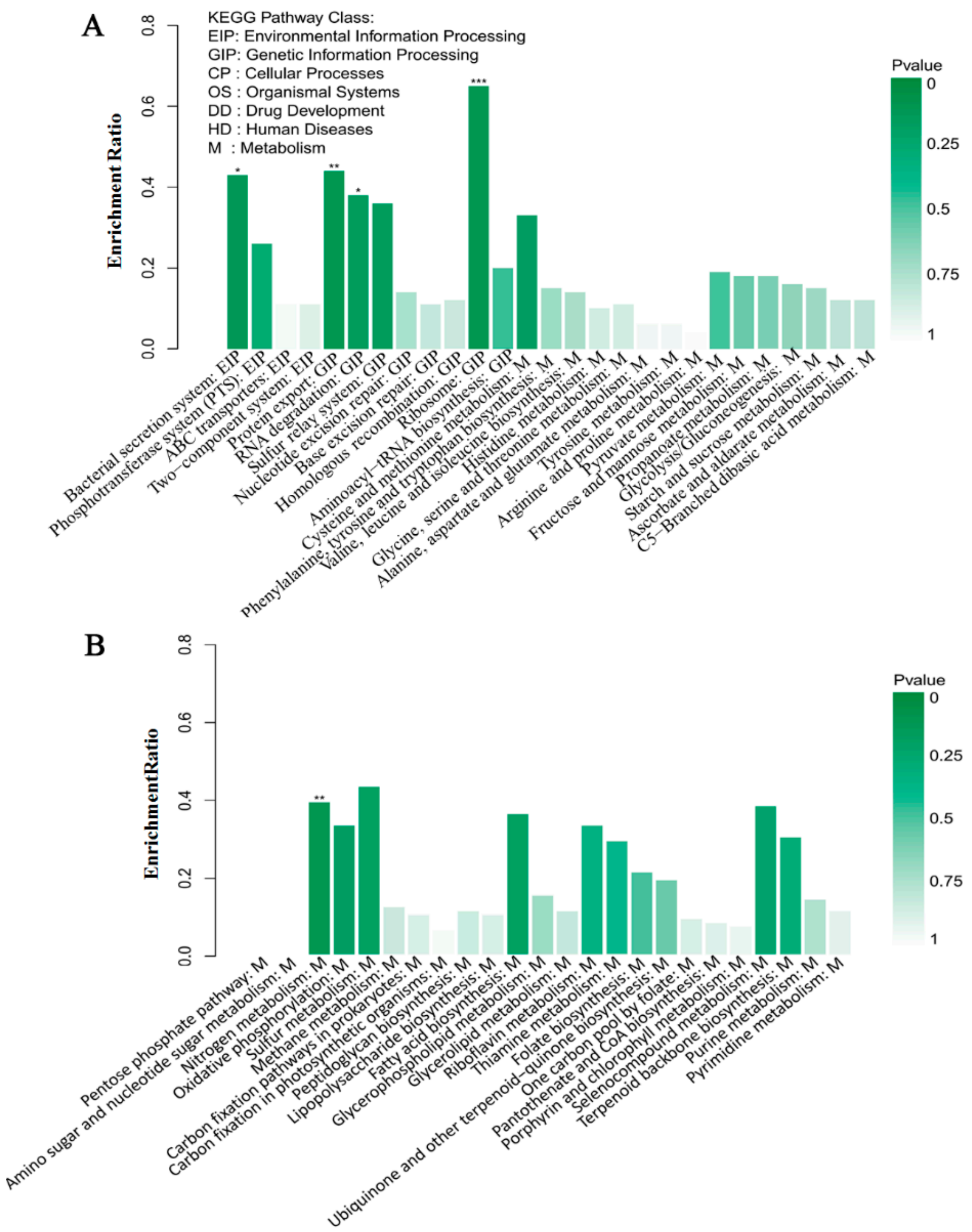

Figure S1. KEGG enrichment analysis of the differentially expressed genes in the Δ*phoP* mutant. KOBAS software was used to analyze the *phoP*-regulated genes in KEGG pathways. Each column indicates one pathway, and the abscissa represents the pathway name and classification. The column color refers to the significance, and the deeper the color the more significant the differences. * $p < 0.05$; ** $p < 0.01$; *** $p < 0.001$.
